# Inflammasomes in Tissue Damages and Immune Disorders After Trauma

**DOI:** 10.3389/fimmu.2018.01900

**Published:** 2018-08-16

**Authors:** Perrine Bortolotti, Emmanuel Faure, Eric Kipnis

**Affiliations:** ^1^Meakins-Christie Laboratories, Department of Medicine, Research Institute of the McGill University Health Center, Montreal, QC, Canada; ^2^Surgical Critical Care Unit, Department of Anesthesiology and Critical Care, Centre Hospitalier Regional et Universitaire de Lille, Lille, France; ^3^Host-Pathogen Translational Research, Faculté de Médecine, Université Lille 2 Droit et Santé, Lille, France

**Keywords:** inflammasome, trauma, DAMP, inflammation, immunosuppression

## Abstract

Trauma remains a leading cause of death worldwide. Hemorrhagic shock and direct injury to vital organs are responsible for early mortality whereas most delayed deaths are secondary to complex pathophysiological processes. These processes result from imbalanced systemic reactions to the multiple aggressions associated with trauma. Trauma results in the uncontrolled local and systemic release of endogenous mediators acting as danger signals [damage-associated molecular patterns (DAMPs)]. Their recognition by the innate immune system triggers a pro-inflammatory immune response paradoxically associated with concomitant immunosuppression. These responses, ranging in intensity from inappropriate to overwhelming, promote the propagation of injuries to remote organs, leading to multiple organ failure and death. Some of the numerous DAMPs released after trauma trigger the assembly of intracellular multiprotein complexes named inflammasomes. Once activated by a ligand, inflammasomes lead to the activation of a caspase. Activated caspases allow the release of mature forms of interleukin-1β and interleukin-18 and trigger a specific pro-inflammatory cell death termed pyroptosis. Accumulating data suggest that inflammasomes, mainly NLRP3, NLRP1, and AIM2, are involved in the generation of tissue damage and immune dysfunction after trauma. Following trauma-induced DAMP(s) recognition, inflammasomes participate in multiple ways in the development of exaggerated systemic and organ-specific inflammatory response, contributing to organ damage. Inflammasomes are involved in the innate responses to traumatic brain injury and contribute to the development of acute respiratory distress syndrome. Inflammasomes may also play a role in post-trauma immunosuppression mediated by dysregulated monocyte functions. Characterizing the involvement of inflammasomes in the pathogenesis of post-trauma syndrome is a key issue as they may be potential therapeutic targets. This review summarizes the current knowledge on the roles of inflammasomes in trauma.

## Introduction

According to the World Health Organization and the Global Burden of Disease study, injuries are responsible for five million deaths per year worldwide (1, 2). Trauma-related mortality is usually described as trimodal: immediate (minutes), early (hours), and late (days to weeks) (3–5). Immediate and early mortalities, mainly due to overwhelming brain injuries, massive bleeding, and critical injuries to vital organs, have remained stable over the past three decades, accounting for approximatively 60 and 25% of overall trauma-related mortality, respectively (2–4). Interestingly, a decline in late deaths has been observed in high-income countries, suggesting that improvement in care is successful to reduce mortality. However, post-trauma care remains challenging, as late mortality still accounts for 10–30% of all trauma-related deaths (3, 5). Trauma patients often develop a “post-injury syndrome,” resulting from an imbalanced systemic reaction to the multiple insults occurring after trauma (6). Initial tissue damage, blood loss and subsequent secondary tissue injuries, lead to the local and systemic release of endogenous mediators acting as danger signals [damage-associated molecular patterns (DAMPs)] (7, 8). Recognition of DAMPs by the innate immune system triggers both an intense pro-inflammatory immune response and a concomitant anti-inflammatory response (9–11). While excessive inflammation promotes the development and propagation of secondary tissue injuries beyond the initial traumatic foci, the anti-inflammatory response leads to host defense impairment and sepsis, contributing altogether to multiple organ dysfunctions, and ultimately, death (12).

**Figure 1:**
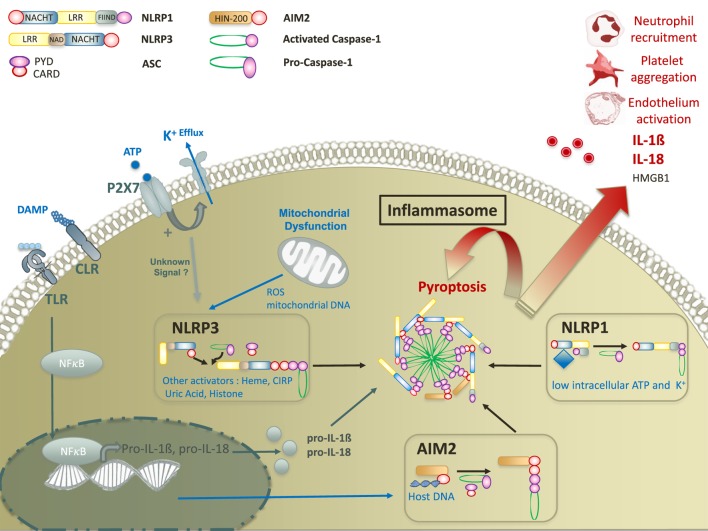
Molecular mechanisms of damage-associated molecular pattern mediated inflammasome activation. Inflammasome activation requires two signals. The first signal ultimately leads to the translocation of NFκB transcription factor to promote the expression of pro IL-18 and pro-IL-1β. The second signal is mediated by a nod-like receptor (NLRP3, AIM2, or NLRP1). Upon activation, NLR activates caspase-1 with or without recruitment of adaptor protein ASC. Activated caspase-1 leads to inflammasome formation and to the secretion of mature forms of IL-18 and IL-1β, and the release of HMGB1. In addition, inflammasomes promote a specific pro-inflammatory cell death, called pyroptosis that contributes to tissue damage in various cellular subtypes. Abbreviations: CIRP, cold-inducible RNA-binding protein; CLR, C-type lectin receptor; ROS, reactive oxygen species; ATP, adenosine triphosphate; TLR, toll-like receptor; IL, interleukine; AIM, absent in melanoma; NFκB, nuclear factor-kappa B; HMGB1, high mobility group box 1.

To date, treatment of the “post-injury syndrome” is limited and mainly supportive. However, a greater understanding of the underlying pathophysiology has led to the development of adjuvant therapeutic strategies. Treatments aiming to control excessive inflammation, monitor immune cell functions, and restore immune responses have emerged, with sometimes encouraging results (9, 11, 13). Among the potential mechanisms involved in the “post-injury syndrome,” inflammasomes could become promising targets. Indeed, some of the trauma-induced DAMPs are recognized by NOD-like receptors (NLRs) leading to the assembly of intracellular multiprotein complexes named inflammasomes (Figure 1). Accumulating data suggest that inflammasomes are involved in the generation of tissue damage as they promote an exaggerated systemic and organ-specific inflammatory response (14). Inflammasome activation in immune cells, endothelial cells, and platelets contribute to microcirculatory dysfunctions and play a critical role in tissue injuries after ischemia and reperfusion (15). Inflammasomes are involved in the development of traumatic brain injury (TBI) (16) and acute respiratory distress syndrome (ARDS) (17). Finally, inflammasomes may also play a role in monocyte dysfunctions involved in post-trauma immunosuppression (18).

In this review, we put into perspective how inflammasomes contribute to the pathophysiology of early post-trauma organ dysfunction, systemically and at specific organ levels. We also discuss the involvement of inflammasomes in the development of post-trauma immunosuppression. Finally, based on existing data from therapeutic strategies targeting inflammasomes in inflammatory diseases, we propose therapeutic perspectives highlighting the challenges of research on inflammasomes in trauma.

## Description and Functions of Inflammasomes

### General Description

Inflammasomes were initially described in 2002 as caspase-1 activating multiprotein complexes (19). Since this seminal report, multiple distinct inflammasomes have been identified. Both pathogen-associated molecular patterns (PAMPs) and endogenous damage-associated molecular patterns (DAMPs) can trigger inflammasome activation. In the context of trauma, we will focus on the inflammasome-mediated response to DAMPs. Inflammasomes are highly expressed in immune cells, especially monocytes and macrophages where they were first discovered. However, inflammasomes are also expressed in a wide range of parenchymal, endothelial (20, 21), and epithelial cell (22, 23) types. Beyond modulation of inflammation, inflammasomes are involved in many physiological processes such as metabolism, cell survival, or tumorigenesis. Consequently, disturbances of inflammasome activity have been involved in a wide range of diseases associated with inflammatory disorders (24). In depth, recent reviews describe inflammasome structures, functions, and regulatory mechanisms providing a translational overview of their role in health and disease, and their potential in development of new immunomodulatory therapeutics (25–30). Here, we provide a brief description of inflammasome biology to introduce their role in post-trauma immune disorders.

### Structure and Functions of Inflammasomes

Inflammasome assembly results from the oligomerization of sensor complexes in response to an intracytoplasmic trigger, allowing procaspase 1 recruitment and activation through interactions of homotypic caspase recruitment domains (CARDs). Depending on the sensor, an adaptor protein known as ASC (apoptosis-associated speck-like protein containing a CARD) is required for caspase recruitment (19). Inflammasomes are named after their intracellular receptor, including NLRs, AIM2-like receptors, RIG-I-like receptors, or pyrin (28). NLRs, the most studied receptors to date, are highly conserved throughout evolution, suggesting an important role in host defense to aggression. NLRs contain an N-terminal signaling domain supporting effector functions, and a C-terminal domain containing leucine rich repeat (LRR) sequences which is usually involved in the interaction with ligands (Table 1). N-terminal and C-terminal domains flank a nucleotide-binding domain that defined NLRs (28) (Figure 1). NLRP3 and NLRP1 inflammasomes may be activated by a wide range of exogenous and endogenous compounds, making them the perfect candidates for inflammasome-mediated inflammatory response in trauma (Table 1) (31, 32). Among non-NLR inflammasomes, absent in melanoma (AIM)2 is one of the most studied. Specifically triggered by cytosolic deoxiribonucleic acid (DNA), AIM2 activation has been reported in trauma. Like NLRP3, activated AIM2 receptor nucleates ASC through PYD–PYD interactions to form AIM2 inflammasome. The structure of NLRP1 slightly differs as it contains both PYD and CARD domains, resulting in the ability to recruit procaspase 1 with or without ASC (33, 34) (Figure 1). Other non-NLR receptors have been shown to assemble inflammasomes, but their roles in critical injury have rarely, if ever, been studied. Of note, some differences in the structural characteristics or mechanisms of assembly have been observed between mouse and human. Thus, the interpretation of results from *in vivo* animal models should always take into account such possible discrepancies (35).

**Table 1:** Inflammasome activators and activated inflammasomes in trauma.

Inflammasome activators	NLRP3	NLRP1	AIM2	Reference
ATP	+			Gombault et al. (36)
K^+^ fluxes	+	+		Pétrilli et al. (37)
Reactive oxygen species	+			Tschopp and Schroder (38)
Uric acid	+			Gasse et al. (39)
Mitochondrial DNA	+			Shimada et al. (40)
dsDNA			+	Hornung et al. (34)
CIRP	+			Yang et al. (41)
Heme	+			Dutra and Bozza (42)

*CIRP, cold-inducible RNA-binding protein; ATP, adenosine triphosphate; AIM, absent in melanoma; DNA, deoxiribonucleic acid*.

### Specific Features of Inflammasomes as Innate Immune Receptors

Like toll-like receptors (TLRs), inflammasomes were recently demonstrated to orchestrate innate immune responses to aggression (25). However, the localizations, mechanisms of activation, and signaling pathways differ between these two pattern recognition receptors (PRRs) (43). TLRs are transmembrane proteins localized either to the cell surface or within endosomes. Thereby, TLRs mainly recognize extracellular compounds *via* their extracellular or intra-endosomal LRR motif (44). Conversely, inflammasomes are intra-cytosolic sensors detecting mostly intracellular stimuli (25). Whereas TLRs activation results from binding to well described specific ligands, the triggers for inflammasome activation are more heterogeneous, ranging from the recognition of specific ligands to the sensing of disturbances in the intracellular environment (29). Activation of TLRs mainly leads to a transcriptional immune response *via* either the MyD88/nuclear factor-kappa B, mitogen-activated protein kinase, or TRIF pathways, resulting in the synthesis of pro-inflammatory proteins, cytokines, and type I Interferon (IFN) (45). Conversely, inflammasome activation does not support any direct transcriptional activity but allows the caspase-1 dependent cleavage of pro-interleukine (IL)-1β and pro-IL-18 into mature forms (27). Importantly, inflammasome activation generally requires a priming step allowing the transcription of the inflammasome components genes (46, 47), in which TLRs are critically involved (47). The second signal comes from the detection of an intracellular “danger” by the cytoplasmic sensor (31). Finally, TLR-signaling was demonstrated to promote various forms of programmed cell death such as autophagy, apoptosis, or necrosis, whereas inflammasomes trigger specific caspase-1-dependent pyroptotic cell death (45, 48).

### Inflammasomes: A Double-Edged Sword in Host Defense

Inflammation is an evolutionarily conserved, protective response to harmful stimuli mounted to preserve or restore the integrity of the body. However, the intensity, duration, and compartmentalization of the inflammatory response needs to be tightly regulated. Excessive, extensive, or prolonged inflammation is responsible for secondary damage, as observed during ARDS or trauma (12). Beyond their critical role in detection and control of intracellular pathogens (49, 50), studies suggest that inflammasomes contribute to tissue regeneration through inflammasome-dependent cytokines which promote effective clearance of damaged cells and tissue repair (22, 51). Alongside these beneficial roles, there is evidence that inflammasome activation is also responsible for unbalanced, excessive inflammation (52). Some of the numerous DAMPs released in trauma are known activators of inflammasomes, suggesting the potential role of inflammasome activation in the pathogenesis of trauma-associated immune disorders (10).

## Damp-Mediated Inflammasome Activation in Trauma

The emergence of the concept of danger rather than “non-self” as the trigger of the innate immune response was a turning point in the history of immunology (53). From this perspective, it is not surprising that various pathologic conditions associated with inflammatory disorders, such as trauma, sepsis, or ARDS, share common pathophysiologic features (54, 55). Through the activation of identical PRRs, PAMPs initiate the immune response to infection whereas DAMPs trigger “sterile inflammation” (10). Conversely to PAMPs that are exogenous compounds of infectious origin, DAMPs are mostly endogenous self-molecules reflecting the alteration of cellular integrity. This explains why some authors proposed the term “alarmin” to specifically designate damage-associated endogenous compounds and broaden the DAMP acronym to “danger-associated molecular pattern” including both alarmins and PAMPs (56, 57). In this review, we chose to use the term DAMP as “damage-associated molecular pattern,” equivalent to alarmin.

The definition and classification of DAMPs are still debated, as any intracellular compound could potentially be a DAMP. DAMPs should be released after cell stress or damage and reflect damage intensity. In addition, DAMPs should trigger an inflammatory response through identified receptors, measurable at physiological concentrations. However, all the endogenous molecules supporting a pro-inflammatory role cannot always be strictly classified as DAMPs based on these criteria (10, 56, 58). Because the nature of DAMPs is extremely heterogenous, and the receptors involved in their recognition are often redundant with other DAMPs or PAMPs, the conceptual framework of DAMPs is not constrained to specific molecular groups or unique signaling pathways. Pragmatically however, the concept of DAMPs provides the opportunity to distinguish exogenous danger signals such as microbial patterns, from endogenous danger signals that can be recognized through innate immune sensors and/or trigger an immune response. Consistently, it has been proposed to take into account the clinical relevance of the compounds in the pathogenesis of the inflammatory response to injury to define DAMPs (10, 56, 58). Indeed, some of these molecules can trigger inflammation through direct interaction with host cells, but also indirectly *via* complement activation for example. Direct interactions with host cells occurs either *via* cell surface and extracellular milieu receptors, such as TLRs or receptor for advanced glycation endproducts (RAGE), or *via* intracellular receptors such as NLRs (10, 59). The intra- versus extracellular compartmentalization of DAMPs determines the nature of activated PRRs (56). In trauma, mechanical tissue injuries and blood loss, associated with secondary events including ischemia/reperfusion (I/R), hypothermia, hypoxia, coagulopathy, or neuroendocrine disorders, lead to cell stress and cell death (9–11). Both cell stress and cell death lead to the active or passive intra- and extracellular release of intracellular compounds that are the major source of DAMPs (56). Several classes of compounds have been identified ranging from the small uric acid or adenosine triphosphate (ATP) molecules to large proteins over 50 kDa. This tremendous structural diversity associated with the extensive variety of PRRs and their mechanisms of interaction highlights the complexity of DAMP signaling, which was previously reviewed (56).

Here, we focus on characterized DAMPs involved in the trauma-related, inflammasome-dependent inflammatory response.

DAMPs are classified depending on their origin, mitochondrial, cytosolic, or nucleic. Mitochondrial DAMPs identified in trauma-induced response are: mitochondrial DNA, reactive oxygen species (ROS), and ATP, which promote NLRP3 inflammasome activation, and NLRP1 inflammasome activation for ATP (38). Cytosolic DAMPs are mostly represented by uric acid, and cold-inducible RNA-binding protein (CIRP), a highly conserved chaperone protein that belongs to the family of cold shock proteins, both of them activating the NLRP3 inflammasome pathway (39, 41). Heme is also reported to activate NLRP3 inflammasome, participating in hemolysis-induced lethality in trauma (42).

Two main nucleic DAMPs are demonstrated to participate in trauma-induced immune response: histones (60) and host DNA (61). Host DNA interacts directly with AIM2 inflammasome, through its HIN domain (46). Histones activate the NLRP3 inflammasome, but the precise pathways are not characterized (60).

The diversity of stimuli described as NLRP3 and NLRP1 activators makes the hypothesis of direct interaction unlikely, but rather supports the existence of common intracellular pathway(s) leading to inflammasome assembly. DAMP-mediated NLRP3 and NLRP1 activation remains poorly understood despite years of research. However, the reduction of intracellular potassium levels through K^+^ efflux channel is recognized as a downstream convergence point (37) (Figure 1).

Interestingly, inflammasome activation could result from extracellular DAMPs released by surrounding stressed or dying cells, or *via* intracellular nucleic and mitochondrial DAMPs translocating into the cytosol in response to cell stress (56).

The nature of these DAMPs and the inflammasomes to which they are related are summarized in Table 1. Importantly, one should keep in mind that other uncharacterized ligands could also be potential activators of the inflammasomes after trauma.

Priming and activation of inflammasomes through DAMPs have been proven in trauma-like models. In proof-of-concept experiments, Iyer et al. show that murine macrophages exposed to necrotic cells produced by pressure disruption, hypoxic injury, or complement-mediated damage, elicit NLRP3 inflammasome priming and activation (62). Likewise, injection of necrotic cells in the peritoneum of wild-type mice leads to NLRP3 activation (62). Consistently, recent studies show the priming and activation of the NLRP3 inflammasome in the injured tissues of rodents in response to mechanical stress (63, 64). Similar observations have been made in the brain, heart, kidney, and testis of mice exposed to hypoxia–ischemia (15). Here, we describe how inflammasome signaling participates in the pathogenesis of remote tissue and organ damage in this context.

## Inflammasomes in the Pathogenesis of Remote Tissue and Organ Damage after Trauma

**Figure 2:**
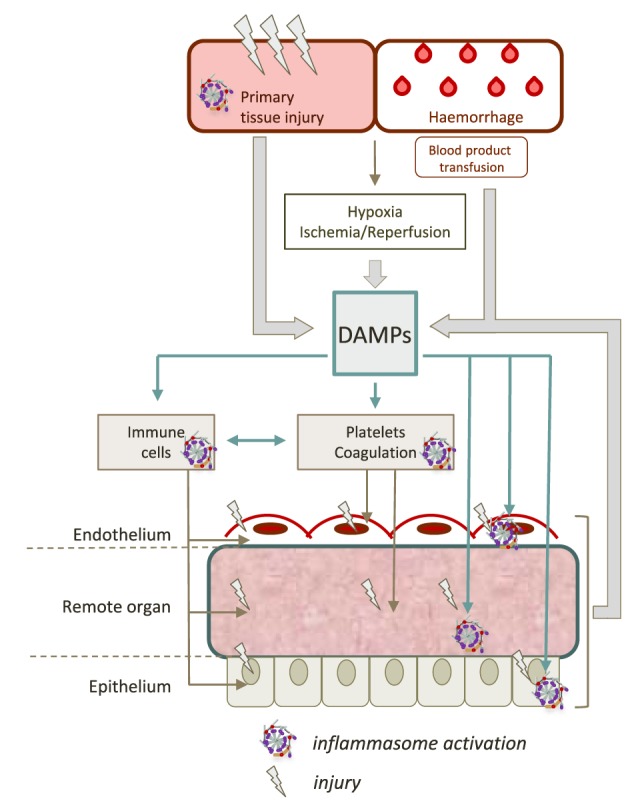
Contribution of inflammasomes to remote tissue damage after trauma. The initial trauma injury induces the release of a large amount of different damage-associated molecular patterns (DAMPs). DAMPs can activate inflammasomes in various cell types such as hematopoietic cells (platelets, neutrophils, monocytes, and macrophages), epithelial cells, or endothelial cells. This dramatic inflammasome activation leads to increase the release of new DAMPs, maintaining a vicious circle, that contributes to worsen organ damage and propagate the pro-inflammatory response.

The pathogenesis of remote organ injury after trauma is a multifactorial process that has already been expertly reviewed (9, 11, 12, 55, 65). The post-trauma period is often associated with an intense pro-inflammatory response termed “systemic inflammatory response syndrome” or SIRS. SIRS often exceeds its functions of clearing and repair, promoting tissue damage independently of the initial injury (12, 55). The concomitant dysfunction of endothelium, coagulation, and immune system promotes the onset and the self-perpetuation of secondary damages to tissues (Figure 2). The extreme expression of this immune deregulation is multiorgan dysfunction syndrome (MODS) which occurs in approximately in one to four trauma patients, and represents the leading cause of “late” death in trauma (66). Because DAMPs both drive and result from tissue damage, they play a central role in the pathogenesis of trauma-related critical illness, supporting a vicious cycle of injury (55). Multiple studies suggest that inflammasome activation, notably NLRP3, is involved in the pathogenesis of SIRS and MODS after trauma.

### Limits to the Study of Host Response Pathophysiology in Trauma

In adult trauma studies, the variability in the timeframes of biological sampling, in patients’ underlying conditions, and in patient management, particularly in the late phase of trauma in which patients are variably addressed to critical care units, intensive care units, step down units, or surgical wards leads to excessively challenging interpretation of results. To address these problems, strictly controlled animal models greatly simplify addressing physiopathological questions, albeit with inherent limitations. The advantages and limitations of animal models of trauma were reviewed by Tsukamoto and Pape (67) and Deitch (68). Regardless of the limitations of animal models, some can provide information even on late phase of trauma and may corroborate clinical observations. For instance, while in adult patients with trauma brain injury, a significant reduction in phagocytosis capacity has been observed more than 3 weeks after the first insult, in a murine brain injury model, a reduced amount of phagocytic cells and a decline of the thymic mass were observed after 2 months post-injury (69, 70). Considering this example, promising data from animal models may be useful even in studying the later phases of trauma and trauma-induced long-term immune disorders.

### Inflammasomes in Uncontrolled Inflammation After Trauma

Both inflammasome-mediated pro-inflammatory mediator release and pyroptotic cell death contribute to initiating, enhancing, and propagating inflammation after trauma (30, 71). In a rat model of acute severe stress, Maslanik et al. demonstrate that caspase-1 activity promotes the production of IL-1β and IL-18 in the circulation and in peripheral tissues (72). In a mouse model of tissue contusion, Starzl et al. show that NLRP3 inflammasome activation is associated with the severity of the inflammatory response through IL-1β and IL-18 secretion (64). Through multiple systemic and local effects, IL-1β is an essential actor of the inflammatory and immune response (73). IL-1β induces the expression of multiple pro-inflammatory genes such as IL-6, IL-8, MCP-1, IL-1α and β, platelet activating factor, and eicosanoids. IL-1β is responsible for the production of acute phase proteins such as CRP and coagulation factors. By enhancing the production of cell surface adhesion molecules and chemo-attractants, IL-1β promotes leukocyte recruitment and activation to the site of inflammation (74, 75). Although less studied than IL-1β, IL-18 also promotes the expression of adhesion proteins, pro-inflammatory cytokines, and chemokines beside its major role as an IFNγ inducer (76). Interestingly, it has been shown that inflammasome activation also plays a critical role in the passive and active extracellular release of high mobility group box 1 (HMGB1), a well-characterized DAMP in trauma (77). HMGB1, a ubiquitously expressed DNA-binding protein located in the nucleus, increases in patients serum and CSF after trauma (78, 79), triggering both pro-inflammatory response and leukocyte recruitment *via* its main receptors RAGE and TLR2/4 (80, 81). HMGB1 is also been involved in post-trauma immunosuppression (82). Therefore, HMGB1 has been proposed as a prognostic biomarker and as a potential therapeutic target in trauma (82, 83).

Together with inflammasome-dependent pro-inflammatory mediators, pyroptosis contributes to the overwhelming inflammatory response. Pyroptosis is characterized by cytoplasm swelling and plasma membrane rupture (84). Pyroptosis allows the destruction of disturbed cells (85), but also leads to the release of the intracellular content into the extracellular space, contributing to “sterile” inflammation. Further inflammasome-dependent mechanisms allow the amplification of the inflammatory burst (27). First, the inflammasome apparatus *per se*, by promoting a large amount of active caspase 1 in response to a single trigger, contributes to amplify the signal (30, 86). Second, inflammasome components themselves, by releasing activated ASC into the extracellular environment, induce further inflammasome activation in surrounding cells (30, 71).

Altogether, inflammasome-mediated inflammation also promotes the recruitment and activation of immune cells through a concentration gradient of DAMPs, cytokines, and chemokines (87). Inflammatory leukocytes are major effectors of post-injury secondary damage (88). When exposed to inflammatory mediators, immune cells start activating. This “priming” step promotes leukocytes adhesion to the microvascular endothelium and their extravasation into tissue through the vascular wall (89). The release by activated leukocytes of a wide array of mediators such as ROS, proteases, cytokines, chemokines, and lipid mediators into ischemic tissues promotes further endothelial barrier and parenchymal tissue damage (90).

### Inflammasomes and Endothelial Dysfunction

Systemic release of DAMPs triggers the diffuse activation of endothelial cells in an organ-specific manner. Endothelial cells do not just form a mechanical barrier but rather constitute an active regulatory organ which plays an essential role in vascular homeostasis and host defense (91). Under physiological conditions, the endothelium continuously maintains an antithrombotic environment by regulating platelet activation, and balancing inhibitors and activators of coagulation and fibrinolysis (91). Under pathological conditions such as trauma, endothelial functions are critical to control hemorrhage by promoting a procoagulant environment while preventing massive thrombosis (92). Vasoconstriction triggered by the decrease in endothelial nitric oxide (NO) production, platelet adhesion, and expression of procoagulant proteins such as endothelial tissue factor, allow the formation of adherent thrombi (Figure 2) (92). The endothelium also regulates the homing and recruitment of leukocytes. Activated endothelium overexpresses cell surface adhesion molecules, chemotactic and activating factors, setting the stage for immune cell recruitment even in non-injured, non-infected remote organs (92). Both clot formation and leukocyte infiltration are mandatory for the control of bleeding and the secondary tissue repair. However, these mechanisms also contribute to worsen, disseminate, and perpetuate injury as well (Figure 2) (92).

**Figure 3:**
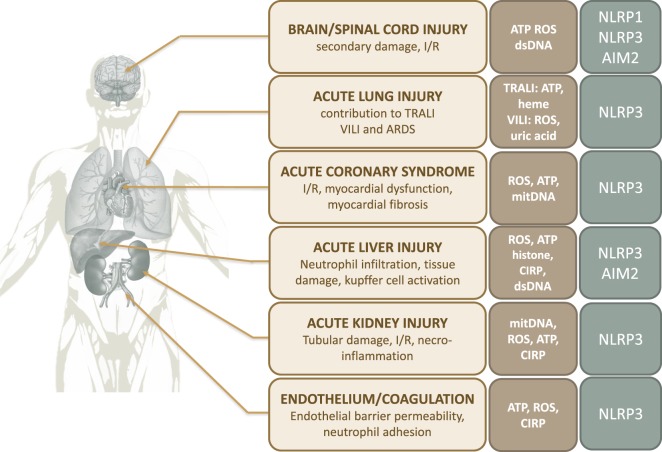
Contribution of inflammasomes to organ damage after trauma. Abbreviations: ARDS, acute respiratory distress syndrome; ATP, adenosine triphosphate; CIRP, cold-inducible RNA-binding protein; I/R, ischemia/reperfusion; ROS, reactive oxygen species; TRALI, transfusion-related acute lung injury; VILI, ventilator-induced lung injury.

There is evidence for the involvement of inflammasomes in endothelial dysfunction, even though specific studies in the context of trauma remain scarce. It has been shown *in vitro* that several DAMPs such as ATP or CIRP activate the NLRP3 inflammasome in endothelial cells (41, 93). The most striking data on the topic arise from studies performed in lung endothelium. Three recent studies using *in vivo* mouse models show that hemorrhagic shock (HS) induces NLRP3 priming and activation in lung endothelial cells, enhancing the pro-inflammatory response through pyroptosis and IL-1β secretion (20, 94, 95). Yang et al. demonstrate that endothelial cell pyroptosis leads to increased inflammation and injury in the lung of mice subjected to a “two-hit” model of HS followed by lipopolysaccharide (LPS). In this model, major neutrophil alveolar recruitment and interstitial edema are attenuated in caspase-1-null mice (94). Xiang et al. find a role for hemorrhage-related ROS production in the activation of endothelial NLRP3 inflammasome, leading to increased levels of IL-1β in BAL (Figure 3) (20). A similar result is found by Xu et al., showing an increase in NLRP3 expression in endothelial cells responsible for higher IL-1β level in bronchoalveolar lavage fluid (20, 94, 95). These inflammasome-mediated responses contribute to endothelial damage associated with vascular leakage, edema, increased leukocyte infiltration, and cytokine release in the lungs (84, 87). Besides their effects on endothelial cells, inflammasomes also modulate other actors of vascular dysfunction and secondary damages, including platelets and coagulation.

### Inflammasomes, Platelet Functions, and Coagulation

Because they are anucleate, platelets were initially considered as simple procoagulant effectors. However, recent research established that platelets also support critical pro-inflammatory and anti-infectious properties beside their role in hemostasis (96, 97). Indeed, activated platelets play a critical role in the inflammatory response, especially in ischemia-reperfusion. Platelets possess various immune receptors, cell surface adhesion molecules, and a broad spectrum of immunomodulatory mediators contained in preformed granules (98). When activated by circulating DAMPs, platelets adhere to endothelial cells and leukocytes to form aggregates. Adherent platelets can either release the content of their granules or synthetize pro-inflammatory mediators (99). Platelets modulate endothelial and immune cell functions. For instance, platelets enhance adhesion molecule expression and chemokine secretion of endothelial cells (98). Most importantly, platelets strongly promote leukocyte activation and adhesion to post-ischemic microvessels, enhancing their recruitment to sites of tissue damage (100). Platelets also directly modulate neutrophil functions such as phagocytosis and degranulation. Thus, platelets play a critical role in reinforcing the pro-inflammatory cycle leading to and perpetuating organ injury (97, 100).

Inflammasomes have been recently shown to be involved in the pro-inflammatory roles of platelets in both infectious and non-infectious diseases (97, 98). The effects of inflammasome activation on platelets, reviewed by Hottz et al., are essentially mediated by the production of IL-1β (97). Briefly, it has been recently discovered that platelets are capable of assembling functional NLRP3 inflammasomes leading to the synthesis of IL-1β (101). Although anucleate, platelets possess a fully functional translational apparatus associated with constitutively present mRNA transcripts, including IL-1β (99, 102). These properties allow rapid synthesis of mature IL-1β after platelet activation (99). Although no study exists in the specific field of trauma, the role of inflammasomes on platelet functions has been studied *in vitro* in models of vascular injury or thrombin-induced platelet activation. These studies show that inflammasome-mediated IL-1β secretion, either in a paracrine fashion or through the release of IL-1β-rich microparticles, activate endothelial cells, with subsequent increase in endothelial permeability, neutrophil adhesion, and endothelial transmigration (97). Aside from their effects on platelet functions, inflammasomes are involved in driving a procoagulant response. Rothmeier et al. show that ATP-driven inflammasome activation in murine bone marrow-derived macrophages promotes the intracellular trafficking of tissue-factor onto the surface of MPs and thus facilitates the release of highly procoagulant MPs (103). These results suggest a deleterious role of DAMP-induced inflammasome activation in the onset of coagulopathy.

### Inflammasome in Ischemia-Reperfusion

Endothelial, platelet, and coagulation dysfunction together with leukocyte activation actively contribute to worsen initial tissue ischemia. While changes in cell physiology under short-acting ischemic conditions represent an appropriate protective process, prolonged or intense ischemia unconditionally leads to cell damage and death (100). In addition, reperfusion of either primary injured or distant ischemic organs is also a critical actor of tissue damage and DAMP production through “ischemia-reperfusion syndrome” (104). At the cellular level, reperfusion triggers massive production of ROS, calcium overload, and severe mitochondrial dysfunction, which are all critical events in the progression of cell death (100). The active contribution of inflammasomes to I/R-induced injuries has been demonstrated *in vivo* using experimental models of mice deficient for inflammasome components or IL-1β and IL-18 signaling pathways. The inflammasomes have been involved in the pathogenesis of injury in the heart, liver, kidneys, central nervous system (CNS), and lungs exposed to I/R.

In the heart, I/R-related phenomenon are responsible for cardiac cell death, enhancing the size and severity of myocardial infarction, and promoting fibrosis after ischemic events (105). The NLRP3 inflammasome has been reported to play a major role in this process *via* IL-1β and IL-18 secretion (95–98) (Figure 3). It has been shown in mouse models of myocardial I/R that inflammasome activation in cardiac fibroblasts (106), cardiomyocytes (107), and cardiac endothelial cells (108) are essential to the inflammatory response driving myocardial I/R injury. Inflammasome activation promotes myocardial inflammatory cell infiltration and increased pro-inflammatory cytokine expression in cardiac tissue. Consistently, myocardial infarct size, contractile dysfunction, and fibrosis are decreased in ASC and caspase-1 deficient mice (106, 109). Likewise, pharmacological inhibition of NLRP3 or IL-1β/IL-18 signaling protects the heart from I/R injury in a rodent model of cardiac ischemia (110–114) (Figure 3). In this context, the role of ROS as activators of the NLRP3 inflammasomes seems predominant (15).

As for the heart, the critical role of inflammasomes is well documented in several acute and chronic human kidney diseases (115). As a result, inflammasome signaling is already an effective therapeutic target for some selected patients with chronic renal failure (115). Inflammasomes are involved in the pathogenesis of acute kidney injury (AKI) related to I/R (15) (Figure 3). With crucial functions in maintaining water and sodium homeostasis, overall plasma electrolyte regulation and detoxification, the kidneys receive more than 20% of the cardiac output. To support their functions, the kidneys and particularly renal tubular epithelial cells have a high mitochondrial density which requires a high continuous energy supply. Thus, they are vulnerable to oxygen and energy deprivation. The release of mitochondrial components, ROS, and other DAMPs from damaged tubular epithelial cells promotes acute cell death (Figure 3). CIRP is also highlighted to participate in I/R-induced damage during AKI, in a murine model using CIRP-KO mice (116) (Figure 3). The subsequent pro-inflammatory burst promotes fluid leakage and parenchymal infiltration of inflammatory cells, worsening renal dysfunction and injury (117). Subsequent tubular necrosis and loss of functional nephrons lead to the clinical syndrome of AKI, explaining the high frequency of acute renal failure in critical illness (118). Once again, DAMPs are the initiators of the inflammatory response. There is substantial evidence that the inflammasome–IL-1β axis plays a key role in the primary mechanisms leading to renal necroinflammation. Macrophages and dendritic cells, in addition to most renal parenchymal cells, express inflammasomes (115). DAMPs released from necrotic cells have been shown to activate the NLRP3 inflammasome in renal tissue. Indeed, Iyer et al. observe an upregulation of *Nlrp3* and *Asc* gene expression in a mouse model of nonlethal renal I/R injury, associated with intense acute tubular necrosis (62). *Nlrp3, Asc*, and *Casp1/11* deficient animals are consistently protected from renal necroinflammation (115, 119). *Nlrp3*-deficiency dramatically improves the survival of animals in lethal renal ischemic injury and provides functional protection against renal failure as suggested by significantly lower plasma urea and creatinine levels. This protection is associated with reduced neutrophil infiltration and IL-1β levels in the renal interstitium of *Nlrp3*-deficient mice (62, 120, 121). The role of IL-1β and IL-18 in acute renal failure is still debated as the results of blockade experiments are controversial (122). Shigeoka et al. find that blocking these cytokines fails to improve creatinine levels and histopathological damage in murine models (120). However, Wu et al. demonstrate that IL-18-null mice show better kidney function, less tubular damage, and reduced necroinflammation. The same observations have been made when mice are pretreated with the IL-18 antagonist IL-18BP (123). Last, NLRP3 might impair tissue repair during the reperfusion phase, as *Nlrp3*-null mice show reduced tubular necrosis and apoptosis together with increased proliferation of tubular epithelial cells after I/R injury (124).

A similar role for NLRP3 and AIM2 inflammasomes has been recently reported in hepatic I/R injury (87, 125, 126) (Figure 3). DAMPs locally released during I/R, namely ROS, ATP, or extracellular histones, have been shown to trigger inflammasomes activation (Figure 3) in Kupffer cells, which critically contribute to the exacerbation of the local inflammatory response (87, 125, 126). Inflammasome-mediated liver injury in I/R arises from mechanisms involving pro-inflammatory cytokines and excessive neutrophil infiltration (127). McDonald et al. and others demonstrate that the I/R-related pro-inflammatory environment leads to the adhesion of circulating neutrophils within liver sinusoids, and facilitates their migration through healthy tissue toward damaged tissue (87). Consistently, both the inhibition of NLRP3 inflammasome activity in mice exposed to liver I/R is associated with decreased levels of cytokines and neutrophil infiltration in hepatic tissue, with a dramatic reduction in histological damage and transaminase release (87, 125, 128, 129). Similar results are found when inhibiting IL-1β signaling *via* blocking antibodies or IL-1-receptor antagonist (87, 127, 128). Finally, blocking inflammasome activators such as CIRP increases the overall survival in a mouse model of hepatic I/R, by decreasing neutrophils recruitment and nitrosative stress level (130).

In addition to their roles in I/R-related injury, inflammasomes are involved in brain and lung dysfunction following trauma.

## Contribution of Inflammasomes to Specific Organ Dysfunctions after Trauma

### Brain Injury

Damage to CNS structures is a major determinant of vital and functional prognosis in trauma patients. Thus, studying the mechanisms leading to CNS injuries following trauma is crucial. While the primary traumatic insult directly leads to immediate tissue damage, the pro-inflammatory innate immune response to injury, termed “neuroinflammation,” is responsible for additional secondary cellular damage and extension of the lesions (131, 132). Among the multiple actors involved in neuroinflammation following trauma, the inflammasomes, specifically NLRP1 and NLRP3, play a major role (133, 134) (Figure 3). Although it has been shown that inflammasome-mediated IL-1β production contributes to CNS tissue repair after trauma (135), the pro-inflammatory response resulting from inflammasome activation, together with glial and neuronal pyroptotic cell death, promote the secondary insult that contributes to worsen and extend initial damage (131). Many cell types, including endothelial cells (21), microglia (136), astrocytes, and neurons (133) are capable of assembling inflammasomes. The role of inflammasomes in TBI and spinal cord injury (SCI) has been recently reviewed (133, 134).

NLRP1 retains a special interest in the context of neurotrauma as a major actor of induction and diffusion of inflammation (16). Importantly, the NLRP1 inflammasome is already preassembled before any stimulation in neurons and other CNS cells (133). It has been proposed that this preassembly of the NLRP1 inflammasome in the CNS may facilitate rapid innate immune response after CNS trauma, or may serve to maintain a constant low level of IL-1β in these cells (133) (Figure 3). CNS NLRP1 activation is triggered by multiple stimuli including the activation of pannexin-1 channels, triggered by high extracellular potassium concentrations (137). Likewise, P2X4 purinergic receptors activated by extracellular ATP induce NLRP1 inflammasome activation (133) (Figure 3).

de Rivero Vaccari et al. report the increased expression of NLRP1 inflammasome, ASC, caspase-1, and subsequent IL-1β in the brain and spinal cord motor neurons following trauma (138, 139). Consistently, Satchell et al. observe that caspase 1 and IL-1β proteins are increased in cerebrospinal fluid in infants and children after severe TBI (140). Moreover, de Rivero Vaccari et al. find that NLRP1 inflammasome proteins are present in exosomes derived from cerebral spinal fluid of patients with SCI and/or TBI (141). These exosomes, by exposing neighboring cells to their cargo of proteins such as IL-1β as well as inflammasome components, contribute to the diffusion of inflammation in the CNS (142).

NLRP3 expression and activation also increase in the brain after direct trauma or following ischemia in rodent models, and NLRP3 knockdown leads to a reduction of brain damage and inflammatory mediators in animal models (143, 144). Consistently, Wallisch et al. report increased NLRP3 levels in CSF from children with severe TBI, which is independently associated with poor outcome (145).

Considering the role of inflammasomes in the particular case of cerebral ischemia, it has been shown that activated inflammasomes, namely NLRP3 and AIM2, increase inflammation, infarct size, and neurovascular damage (144, 146). IL-1β-triggered inflammation is a major contributor to cell death in the ischemic brain, but some studies report inflammasome-mediated effects that are independent of IL-1β production. Consistently, inflammasome inhibition or blockade of IL-1β both significantly decrease neuronal cell death in the brain or spinal cord of ischemic animals (147). The link between inflammasome activity, CNS inflammation, and functional outcome leads some authors to suggest the use of inflammasome protein levels in cerebrospinal fluid of brain-injured patients as biomarkers of functional prognosis (148). Interestingly, regulatory mechanisms that downregulate NLRP1 or NLRP3 activity exhibit a protective role against CNS injury (133, 134). For example, Lin et al. show that the downregulation of NLRP1 activity by heme oxygenase-1 decreases NLRP1 inflammasome-induced neuronal death and improves functional recovery in a rat model of spinal cord compression (149). Experimental inhibition of inflammasomes in rodent models of TBI or SCI, targeting either the inflammasome components ASC or NLR, caspase-1 or IL-1β, shows promising results in term of histopathological improvement and improved functional outcome (132, 138, 149–153) (Figure 3).

However, in TBI, inflammasome activation is not the only driver of organ dysfunction. It has indeed been shown that the CNS, particularly through the Hypothalamic–Pituitary–Adrenal axis, drives control of the peripheral immune response (154). This topic has been previously extensively reviewed (155, 156). In brief, the studies suggest that CNS injury impacts both circulating immune cells populations and function, potentially contributing to secondary damage to remote organs and susceptibility to infection (88, 157).

### Acute Lung Injury

The lung is a vital organ supporting blood oxygenation and decarboxylation necessary to aerobic life. Gas exchange between blood and air is highly dependent on the integrity of the alveolar-capillary membrane. Any event leading to tissue damage may impair oxygenation and compromise survival. Thus, lung immune responses and inflammatory processes have to be tightly regulated to deal with aggression while maintaining lung structure and homeostasis compatible with respiratory function. The lungs are exposed through the pulmonary vessels to central venous blood conveying systemic DAMPs from injured tissues. Thus, the lungs are highly susceptible to developing innate responses leading to acute lung injury in response to systemic inflammation encountered in critical illness. Accumulating evidence in the literature suggests that inflammasome-dependent excessive inflammation is involved in the pathogenesis of acute lung injury (158, 159).

It has been shown that trauma-related acute respiratory conditions lead to systemic and local NLRP3 inflammasome activation. Dolinay et al. report the increase of caspase 1, IL-1β, and IL-18 mRNA and subsequent IL-18 and caspase 1 protein levels in peripheral blood of patients with trauma-related ARDS (160). Other teams report NLRP3 inflammasome expression and activation in the lungs of mice undergoing I/R acute lung injury (20, 95, 159) (Figure 3). Various conditions encountered in trauma, such as HS, lung contusion (150, 151), burns (161), ventilator-induced lung injury (VILI), or transfusion-related acute lung injury (TRALI), have been shown to activate the NLRP3 inflammasome in the lung. Animal studies report NLRP3 inflammasome activation in lung endothelial cells and alveolar macrophages (AMs) after HS, resulting in the amplification of local inflammation and IL-1β secretion (20, 94, 95). Consistently, inhibition of the NLRP3 inflammasome attenuates acute lung injury, attested by the decrease in histopathologic damages, reduction in myeloperoxidase activity and inflammatory cytokines in lung tissue (161) (Figure 3).

Inflammasomes have also been implicated in the development of VILI. VILI is caused by lung overinflation during mechanical ventilation (MV) responsible for baro- and volo-trauma that induce overwhelming inflammation (162) (Figure 3). Recent studies demonstrate that sterile inflammation in response to MV is NLRP3-dependent. In mice, MV increases inflammasome gene expression in lung tissue and AMs (160, 163–165). Concomitantly, MV enhances IL-Iβ and L-18 protein levels in the lung and bronchoalveolar lavage. Genetic deletion of *Nlrp3, Caspase-1*, or *Il-18* is associated with reduced MV-induced lung injury, assessed by alveolar neutrophil infiltration, alveolar-interstitial edema, BAL fluid protein content, and pulmonary cell apoptosis (160). Likewise, IL-18 or IL-1β neutralizing antibodies significantly reduce MV-induced inflammatory lung injury. The authors show that AMs are the main cell type involved in IL-1β and IL-18 production (160). Though the exact mechanisms remain to be completely elucidated, it is hypothesized that biomechanical cell trauma related to MV may lead to the release of intracellular DAMPs inducing inflammasome activation (163). Indeed, Wu et al. show that NLRP3 inflammasomes are activated in mouse AMs exposed to cyclic stretch *in vitro*. They report that mitochondrial generation of ROS, together with uric acid release, are responsible for stretch-induced NLRP3 inflammasome activation (164). Consistently, Kuipers et al. find increased uric acid levels in BAL fluid of ventilated mice (163). Finally, patients suffering from ARDS also show increased IL-18 concentration in the serum, whose levels were correlated with lactate concentration, APACHE II score, and mortality (160).

Last, inflammasomes may play a role in the pathogenesis of TRALI (Figure 3). Patients with severe trauma often require blood transfusions. TRALI is a rare but severe complication occurring within 6 h of blood transfusion (166). The pathogenesis of TRALI remains unclear, but might arise from the injury of the pulmonary microvasculature in a “two-hit” model (167). The “first hit” or priming step is an initial injury to the pulmonary endothelium that promotes endothelial cell activation. Mechanisms of this initial pulmonary endothelial injury may be as diverse as MV, direct traumatic injury, and/or sepsis. Endothelial activation leads to adherence, activation, and sequestration of neutrophils in the pulmonary capillary beds. The “second hit” is mediated by the blood transfusion itself. Indeed, blood storage is associated with a varying degree of hemolysis, which releases DAMPs into the stored blood units. The second exposure of the primed lung circulation to DAMPs is thought to further activate neutrophils, triggering the intense pro-inflammatory response that leads to acute lung injury (167). Some authors propose that the NLRP3 inflammasome plays a role in this inflammatory process, linking the two-hit pathogenesis of TRALI with the two-step activation of inflammasomes (167). Indeed, the priming step of TRALI may coincide with the expression of NLRP3 inflammasome in the different types of immune and endothelial cells in the lung, while the second step involving DAMPs such as heme or extracellular ATP, may trigger inflammasome activation and subsequent inflammation (168). However, this hypothesis remains to be confirmed (167).

## Impairment of Inflammasome Functions as a Component of Trauma-Induced Immunosuppression

After severe injury, an anti-inflammatory response named compensatory anti-inflammatory response syndrome (CARS), occurs concomitantly to the pro-inflammatory response. When excessive or persistent, CARS leads to the severe systemic anti-inflammatory response syndrome, which promotes immunosuppression, secondary infections, and late or persisting organ dysfunctions (12, 169). The deactivation of monocytes/macrophages is an important component of immunosuppression following trauma (157–160). Interestingly, inflammasomes are major effectors of monocyte/macrophage immune functions. Although no direct proof exists, two studies suggest that inflammasome function impairment in immune cells may be involved in post-trauma immunosuppression.

Relja et al. show that NLRP1 gene expression after LPS stimulation is reduced in monocytes isolated from trauma patients compared to healthy volunteers (18). The decrease in mRNA levels of NLRP1 exists upon admission to the emergency department and persists over 10 days of immune monitoring. Although NLRP1 protein levels are not assessed in this study, the release of IL-1β from monocytes of trauma patients decreases but is restored with NLRP1 transfection. The authors hypothesize that restoring NLRP1 activity may improve the immune response to PAMPs/DAMPs in this context. However, the role of the other critical inflammasomes such as NLRP3 is not concomitantly assessed (18). In a non-trauma study that enrolled 51 patients who had undergone cardiopulmonary resuscitation after cardiac arrest, the levels of AIM2 gene expression and activity are downregulated in monocytes of resuscitated patients compared to control patients (170). Conversely, *NLRP3, ASC*, and *IL-1β* are upregulated at 12, 24, and 48 h following cardiac arrest. Interestingly, a time-dependent decrease in monocyte NLRP1, NLRP3, ASC, and IL-1β mRNA expression levels is observed in patients who die during the first 30 days after CPR, whereas survivors have stable expression of these transcripts over time. Non-survivors at 30 days have significantly lower mRNA levels of NLRP3 and CASP1 48 h after return of spontaneous circulation following resuscitation, with the same trend observed for NLRP1. The authors also find a reduced ability to release IL-1β in whole blood and monocytes isolated from patients after CPR (170). The impaired systemic IL-1β response of leukocytes from trauma patients was already observed prior to the discovery of inflammasomes although they were yet to be supported by a physiological explanation. Indeed, a decrease in IL-1β protein levels in a group of 14 trauma patients compared to healthy controls was observed until day 5 post-trauma (171). Likewise, other studies on the early inflammatory response in multiple-trauma patients found that blood IL-1β levels in post-trauma patients weakly increased compared to other pro-inflammatory cytokines like tumor necrosis factor α or IL-6 (172). At the time, it was therefore concluded that IL-1β was not a good candidate in the search of potential early predictive markers for systemic inflammatory response after trauma (172).

The impairment of inflammasome activation found in trauma patients is consistent with the data observed in the post-septic immunosuppression syndrome. Alterations in NLRP1 mRNA expression are detectable in human monocytes isolated from patients with septic shock and seem associated with monocyte deactivation. Monocytes from patients in the early phase of septic shock show alterations of caspase-1 gene expression. Also, relative mRNA copy numbers for ASC, caspase-1, and NLRP1 are significantly lower. Finally, NLRP1 mRNA levels are linked to survival in patients with sepsis and correlated with SAPS II scores. In accordance with the observations made in trauma patients, these data suggest that impairment of inflammasome functions in immune cells occurs during the earliest stages of the illness and are involved in monocyte deactivation (173).

The dampening of inflammasome signaling in circulating immune cells is the opposite of what appears to happen in tissues and organs. This is similar to discrepancies reported in the cytokine profiles between blood and injured organs in post-injury patients. Most studies show that IL-1β level increases in damaged tissue while remaining stable or decreasing in blood (156). This illustrates the challenging nature of dynamic host response monitoring, beginning with the selection of the best clinical samples in which to assess the immune response. Whole blood samples are readily and repeatedly accessible and allow characterization of circulating immune cell populations and activation through increasingly clinically available flow cytometry. However, due to the compartmentalization of inflammation in severe injury (156, 174), the information obtained from whole blood may not be extrapolated to injured tissues/organs.

Whether the impairment of inflammasome signaling in monocytes of severely injured patients plays a role in the post-trauma immunosuppression phenotype or only represents a biomarker for severity or immune impairment is uncertain. Data focusing on the involvement of inflammasomes in preserving an effective immune response against secondary infection have yet to be provided.

Some of the treatments that have been evaluated in the post-trauma immunosuppression syndrome interfere with the inflammasome pathway. Glucocorticoids (GC) have long been known as anti-inflammatory compounds. However, an increasing amount of studies suggests that GC also support a pro-inflammatory role through inflammasome priming. GC have been proposed as an important trigger for neuroinflammation through NLRP3/NLRP1 inflammasome priming in microglial cells (175). Moreover, GC induce NLRP3 expression in human macrophages *in vitro*, sensitizing cells to inflammasome triggers, and facilitating inflammasome-mediated release of pro-inflammatory molecules (176). Although no evidence supports this hypothesis to date, the effect of GC-induced inflammasome priming may be a potential mechanism for the anti-VAP effect of GC in severe trauma patients (177, 178), as the role of macrophages and IL-1β in host defense against respiratory infections is well known (179).

Likewise, IFNγ and GM-CSF therapies have been proposed to restore the functions of immune cells after critical injury through HLA-DR induction, especially in a selected population of patients with slow mHLA-DR recovery (180, 181). There is evidence in the literature suggesting that both IFNγ and GM-CSF modulate inflammasome signaling. The role of IFNγ in inflammasome activation has been reviewed recently and remains ambiguous (182). On one hand, it has been shown that IFNγ could upregulate NLRP3 components expression (182). On the other hand, IFNγ has been shown to indirectly inhibit NLRP3 assembly and activity *via* iNOS induction and NO production. Consistently, iNOS^−/−^ mice show enhanced NLRP3 activity and higher mortality in a model of LPS-induced sepsis (183). A few studies report that GM-CSF enhances NLRP3 activity and IL-1β production by macrophages. Shaw et al. show that monocyte/macrophages from GM-CSF-neutralized mice produce less IL-1β *in vivo* and *ex vivo*, together with a decreased expression of NLRP3, pro/active IL-1β, and pro/active caspase 1. Consistently, *in vitro*-derived GM-CSF-differentiated macrophages express higher levels of NLRP3, caspase 1, and IL-1β compared to M-CSF-differentiated macrophages (184). Similar results have been found when studying the inflammasome activity in human monocyte-derived macrophages differentiated in the presence of GM-CSF *in vitro* (185, 186).

Taken together, these data may suggest some potential inflammasome-mediated mechanisms of GC, IFNγ, and GM-CSF to prevent secondary infections in post-injury immunosuppression and highlight the need for in-depth understanding of the underlying pathophysiology to guide the development of targeted, personalized therapies.

## Future Perspectives and Concluding Remarks

When considering another deregulated immune response in trauma, trauma-induced coagulopathy, one can realize how deeply deciphering its pathophysiology has changed the management of trauma care (24). Trauma-induced coagulopathy is now routinely modulated as early as prehospital care through the prophylactic administration of tranexamic acid to counter early hyperfibrinolysis, is monitored throughout patient care using routine laboratory exams or point-of-care thromboelastography/metry, and is treated with a better understanding of transfusion thresholds and beneficial blood product ratios (187). These improvements in the early management of trauma care stem from decades of deciphering a complex innate immune response that goes far beyond clot formation and clearance (188).

Likewise, we have yet to fully understand the later phase of some trauma patients who develop organ failures and/or secondary infections leading to sepsis, both responsible for late trauma deaths. The emerging characterization of trauma-induced immunosuppression seems to be a major underlying mechanism of such late phase morbidity and mortality (189).

Given the roles of the inflammasomes we have reviewed above in generating the initial inflammatory burst and/or amplifying inflammatory responses, the inflammasomes are potentially key actors involved in late trauma organ failure and trauma-induced immunosuppression. Early post-trauma inhibition of inflammasome activation, by preventing pyroptosis-related DAMP release and IL-1β production in damaged tissues, may help to break the vicious cycle of injury propagation and may be useful as an early biological damage-control therapy (9, 156). Likewise, such strategies may be interesting in preventing secondary, DAMP-mediated tissue damage and remote organ injury (156). These hypotheses remain to be proven clinically as the majority the studies are based on animal or *in vitro* experimental models.

Inhibitors of inflammasome signaling already exist; some of which are commercially available and successfully applied to other diseases. Most of these treatments target IL-1β or IL-18: recombinant IL-1R antagonist (anakinra), IL-1β blocking antibody (canakinumab), IL-1 receptor soluble decoy (rilonacept), and IL-18-binding protein soluble receptors or blocking antibodies. Such treatments have transformed patient management, prognosis and quality of life in (auto)inflammatory diseases such as gout, type I diabetes or hereditary disorders like cryopyrin-associated autoinflammatory syndrome (CAPS) (190). The recent discovery of small molecule inhibitors selectively targeting the NLRP3 inflammasome rather than IL-1β might be promising for future research (191, 192). As pointed out by the authors, the potential benefits of these molecules would be increased therapeutic potential through the simultaneous blockade of IL-1β, IL-18, and pyroptosis, and a better safety profile, by only inhibiting NLRP3-dependant IL-1β and IL-18 production while preserving their secretion by other inflammasomes or redundant pathways against infection (191).

Indeed, the essential role of inflammasomes in antimicrobial defense raises the issue of potentially increasing the risk of secondary sepsis when interfering with inflammasomes. The prospective follow-up of CAPS patients treated with anakinra shows a median yearly rate of 7.7 adverse events per patient, among which most of the severe adverse events are infections (193). Likewise, a systematic review finds that 129 (5.1%) severe infections were reported in 2,896 patients treated with anakinra (194). Conversely to its effect in such chronic diseases, the role of anakinra in severe acute infections may be less clear due to concomitant potential beneficial effects in sepsis. Indeed, although anakinra in sepsis yields no benefit on primary outcomes in a historic phase III trial (195), a *post hoc* data reanalysis shows that it significantly improves in survival of a subgroup of patients with sepsis and concurrent features of hepatobiliary dysfunction/disseminated intravascular coagulation similar to macrophage activation syndrome (196). Considering that both SIRS and CARS severity are related to the intensity of the initial trigger as suggested by an increasing number of studies (181, 197, 198), early inhibition of the inflammasome activity may also prevent/decrease the intensity of the post-trauma immunosuppression by attenuating initial biological damage (199).

In their recent review on the immune response to trauma, Huber-Lang et al. provide an excellent overview of the promising strategies that are currently evaluated to treat the post-trauma syndrome (9). As debated above, the constant improvement in the comprehension of the post-trauma syndrome pathophysiology enables the development of pioneering strategies which belong to the field of precision medicine. In a break with past treatments that aimed to dampen or enhance the immune response (GC, prostaglandin, aprotinin, pro immunonutrition, etc.) (180), current approaches are directed toward the main actors of the innate immune response involved in the early “biological damage,” including DAMPs, complement, coagulation, glycocalyx, macrophages, and neutrophils. Such strategies could be considered as “preventive” rather than “curative” for the treatment of SIRS, CARS, and PICS. In addition, personalized evaluation of the immune profile combined with “boosting” immunotherapies are currently assessed for the management of the post-trauma immunosuppression. Because they might contribute to both post-trauma biological damage and immunosuppression, inflammasome represents a relevant pathway to explore in these perspectives.

This review is in favor of a trauma research framework at the interface with a relatively recent field of immunology that may lead to the characterization of a trauma-induced inflammasomopathy and perspectives for modulation with an impact on patient outcomes that remain to be determined.

## Author Contributions

PB, EF, and EK conceived and designed the review. PB wrote the first draft of the manuscript. PB, EF, and EK wrote sections of the manuscript. EF conceived and drafted the figures. EK, PB, and EF provided critical revisions to the final manuscript.

## Conflict of Interest Statement

The authors declare that the research was conducted in the absence of any commercial or financial relationships that could be construed as a potential conflict of interest.

## Abbreviations

AIM, absent in melanoma; AKI, acute kidney injury; ALR, AIM2-like receptor; ASC, apoptosis-associated speck-like protein containing CARD; ARDS, acute respiratory distress syndrome; ATP, adenosine triphosphate; BALF, bronchoalveolar lavage fluid; CARD, caspase recruitment domain; CARS, compensatory anti-inflammatory response syndrome; CIRP, cold-inducible RNA-binding protein; CLR, C-type lectin receptor; CNS, central nervous system; DAMP, damage-associated molecular pattern; DNA, deoxiribonucleic acid; HMGB1, high mobility group box 1; HS, hemorrhagic shock; IFN, interferon; IL, interleukine; I/R, ischemia/reperfusion; LPS, lipopolysaccharide; MODS, multiorgan dysfunction syndrome; MV, mechanical ventilation; NFκB, nuclear factor-kappa B; NLR, nod-like receptor; NLRC, nucleotide-binding oligomerization domain, Leucine rich repeat and caspase domain containing; NLRP, nucleotide-binding oligomerization domain, Leucine rich repeat and pyrin domain containing; NOD, nucleotide oligomerization binding domain; PAMP, pathogen-associated molecular pattern; PRR, pattern recognition receptor; PYD, pyrin domain; RAGE, receptor for advanced glycation endproducts; RLR, RIG-I-like receptor; RNA, ribonucleic acid; ROS, reactive oxygen species; SARS, systemic anti-inflammatory response syndrome; SCI, spinal cord injury; SIRS, systemic inflammatory response syndrome; TBI, traumatic brain injury; TLR, toll-like receptor; TNF, tumor necrosis factor; TRALI, transfusion-related acute lung injury; VILI, ventilator-induced lung injury.
